# Distinct Metabolites for Photoreactive l-Phenylalanine Derivatives in *Klebsiella* sp. CK6 Isolated from Rhizosphere of a Wild Dipterocarp Sapling

**DOI:** 10.3390/molecules18078393

**Published:** 2013-07-16

**Authors:** Lei Wang, Wataru Hisano, Yuta Murai, Munenori Sakurai, Yasuyuki Muto, Haruka Ikemoto, Masashi Okamoto, Takashi Murotani, Reika Isoda, Dongyeop Kim, Yasuko Sakihama, Irnayuli R. Sitepu, Yasuyuki Hashidoko, Yasumaru Hatanaka, Makoto Hashimoto

**Affiliations:** 1 Division of Applied Bioscience, Graduate School of Agriculture, Hokkaido University; Kita 9, Nishi 9, Kita-ku, Sapporo 060-8589, Japan; 2 Forest Microbiology Laboratory, Forest and Nature Conservation Research and Development Center, Forest Research and Development Agency, Jalan Gunung Batu No.5, P.O. Box 165, Bogor 16610, Indonesia; 3 Graduate School of Medicine and Pharmaceutical Sciences, University of Toyama, 2630 Sugitani, Toyama 930-0194, Japan

**Keywords:** photoaffinity label, phenylalanine, tryptophan, phenyl azide, benzophenone, trifluoromethyldiazirine, metabolites

## Abstract

Photoaffinity labeling is a reliable analytical method for biological functional analysis. Three major photophores—aryl azide, benzophenone and trifluoromethyldiazirine—are utilized in analysis. Photophore-bearing l-phenylalanine derivatives, which are used for biological functional analysis, were inoculated into a *Klebsiella* sp. isolated from the rhizosphere of a wild dipterocarp sapling in Central Kalimantan, Indonesia, under nitrogen-limiting conditions. The proportions of metabolites were quite distinct for each photophore. These results indicated that photophores affected substrate recognition in rhizobacterial metabolic pathways, and differential photoaffinity labeling could be achieved using different photophore-containing l-phenylalanine derivatives.

## 1. Introduction

**Scheme 1:**
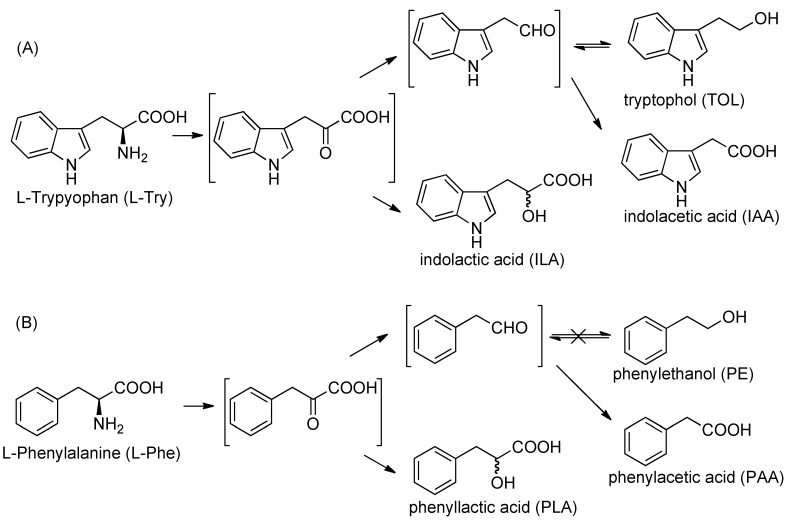
Metabolic pathway for l-tryptophan and l-phenylalanine inoculated into *Klebsiella* sp. CK6 under nitrogen-limiting conditions.

Photoaffinity labeling is a useful biochemical method used to reveal structural and functional relationships between low molecular weight bioactive compounds and biomolecules [1,2,3,4,5,6], and, for this purpose various photophores, such as phenyl diazirine, phenyl azide and benzophenone, are commonly used. Although comparisons of the chemical and physiological properties of these photophores have been reported [7], there are no reports concerning comparative analysis of metabolites resulting from inoculation of these photophores contained in precursors. A rhizobacterium, *Klebsiella* sp. CK6, which was isolated from a dipterocarp sapling in Central Kalimantan, Indonesia, is able to grow in a low-nitrogen medium supplemented with l-tryptophan [8]. Some bacteria use transaminase for the oxidative deamination reaction of l-tryptophan to yield indol-3-acetic acid (IAA) via intermediary indole-3-pyruvic acid [9]. Supplementation of l-tryptophan into the culture medium of *Klebsiella* sp. CK6 afforded IAA, tryptophol (TOL), and indole-3-lactic acid (ILA) under nitrogen-limiting conditions [Scheme 1(A)]. However, the other aromatic α-amino acids were not tested as nitrogen sources. Phenylalanine is one of the most fundamental aromatic α-amino acids and plays an important role in hydrophobic interactions responsible for substrate binding to biomolecules. Photoreactive phenylalanine derivatives can be applied to the biological functional analysis of α-amino acids and bioactive peptides instead of phenylalanine. These strategies are premised on the fact that photoreactive phenylalanine derivatives act in almost the same way as phenylalanine itself. Currently, there are no reports that analyze comparatively the metabolites of photoreactive precursors inoculated into microorganisms. In this paper, we report that l-phenylalanine acts as nitrogen source, but the resulting metabolites are slightly different from those of l-tryptophan, and each photoreactive l-phenylalanine derivative affords different proportions of metabolites in *Klebsiella* sp. CK6 under nitrogen-limiting condition.

## 2. Results and Discussion

### 2.1. Inoculation of l-phenylalanine as a Nitrogen Source for Klebsiella sp. CK6

l-phenylalanine as nitrogen source was inoculated into *Klebsiella* sp. CK6 in a standing culture at 28 °C for one week under nitrogen-limiting conditions, and cell growth did not change compared to when the l-tryptophan was used as nitrogen source. The inoculate mixture was centrifuged to remove planktonic bacterial cells, and the pH of the supernatant was adjusted to 2.5 with 1N HCl. Then, the metabolites were extracted with ethyl acetate. After drying over MgSO_4_ and filtration, the organic layer was concentrated to afford crude extracts. The crude extracts were subjected to silica column chromatography. Phenyllactic acid (PLA) and phenylacetic acid (PAA) were the major metabolites from l-phenylalanine supplemented at pH 6. However, phenylethanol (PE), which is deaminated, decarboxylated, then reduced, was not detected, even though tryptophol (TOL), which undergoes a similar sequence after l-tryptophan inoculation, was easily detected [Scheme 1(B)]. These results indicated substrate specificity, since the reduction from aldehyde to alcohol recognized the indole ring. D-Phenylalanine was less metabolized to PLA and PAA as compared to the l-isomer. No substantially different metabolites were observed after one week under pH conditions between 5 and 6, with or without rotation of the l-phenylalanine inoculum. These results indicated that the metabolic pathways of l-tryptophan and l-phenylalaninewere slightly different due to their chemical structures. 

### 2.2. Inoculation of Photoreactive l-phenylalanine Derivatives for Klebsiella sp. CK6

#### 2.2.1. Inoculation of 4-Azido-l-phenylalanine as a Nitrogen Source for *Klebsiella* sp. CK6

**Scheme 2:**
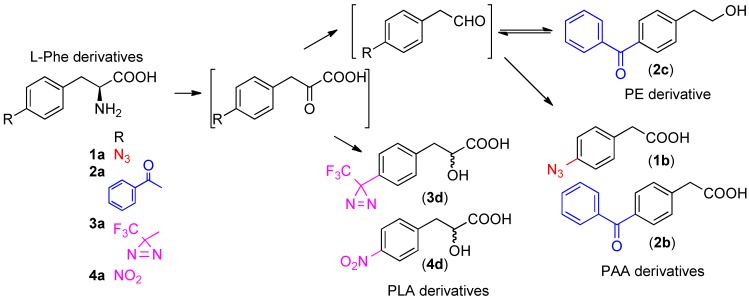
Metabolic pathway for 4-photophore-substituted and 4-nitro-substituted phenylalanine derivatives inoculated into *Klebsiella* sp. CK6.

4-Azido-l-phenylalanine (**1a**) was prepared in a similar manner as described by Wieland *et al.* with some slight modifications [10]. Briefly, phenylalanine was nitrated with nitric acid (fuming) in sulfuric acid. The 4-nitro derivative was reduced by hydrogenation with Pd-BaSO_4_ to afford the aniline derivative. Diazotization, followed by azidation, afforded the target 4-azido-l-phenylalanine in moderate yield. Inoculation of the 4-azido-l-phenylalanine into *Klebsiella* sp. CK6 in the standing culture afforded an azide-substituted PAA derivative **1b** as the main metabolite. IR measurements of the main product indicated that the azide group (2,140 cm^−1^) persisted after inoculation. No reduction of the azide to an amine or other functional group was observed under direct culture analysis (Scheme 2).

#### 2.2.2. Inoculation of 4-benzoyl-l-phenylalanine as a Nitrogen Source for *Klebsiella* sp. CK6

4-Benzoyl-l-phenylalanine (**2a**) was prepared by Friedel-Crafts benzoylation of l-phenylalanine with benzoyl chloride in trifluoromethanesulfonic acid (TfOH). Direct Friedel-Crafts benzoylation of l-phenylalanine has not been reported, presumably due to the low solubility of unprotected α-amino acids in many organic solvents. The solubility of α-amino acids and the properties of the Friedel-Crafts reaction catalyzed by TfOH enabled us to directly benzoylate l-phenylalanine. The inoculation of 4-benzoyl-l-phenylalanine into *Klebsiella* sp. CK6 in standing culture afforded benzoyl-substituted PAA and phenylethanol (PE) derivatives **2b** and **2c** as the main metabolites. No PLA derivative was detected in the extract (Scheme 2). 

#### 2.2.3. Inoculation of 4-[3-(Trifluoromethyl)-3*H*-diazirin-3-yl]-l-phenylalanine as a Nitrogen Source for *Klebsiella* sp. CK6

4-[(3-Trifluoromethyl)-3*H*-diazirin-3-yl]-l-phenylalanine (**3a**) was prepared in an asymmetric manner from corresponding diazirinyl benzyl bromide, which was consistent with our previous reports [11,12]. The inoculation of 4-(3-trifluoromethyl)-l-phenylalanine into *Klebsiella* sp. CK6 in standing culture afforded trifluormethyldiazirinyl-substituted PLA **3d** as the sole product. The UV spectrum of the isolated metabolite indicated a broad adsorption around 350 nm, suggesting that the diazirinyl photophore was still intact. No optical rotation was observed for the metabolite. PAA and PE type metabolites were not detected in the extract (Scheme 2). 

#### 2.2.4. Inoculation of 4-nitro-l-phenylalanine as a Nitrogen Source for *Klebsiella* sp. CK6

4-Nitro-l-phenylalanine (**4a**), which was a synthetic intermediate for the preparation of 4-azide-l-phenylalanine, was fed to *Klebsiella* sp. CK6 to reveal a substitution effect for the three photophores. The nitrated PLA derivative **4d** was identified as the sole metabolite, similar to that of 4-[3-(trifluoromethyl)-3*H*-diazirin-3-yl]-l-phenylalanine. No optical rotation was observed for the metabolite. These results indicated that electron-withdrawing substitution at the 4-position promoted the formation of PLA-type compounds as major metabolites (Scheme 2). 

The electron-donating group at the 4-position of the phenyl ring (azide and benzoyl) promoted stabilization of the α-keto acid skeletons by their conjugations via the phenyl ring, and decarboxylation (phenylacetaldehyde) was preferred over reduction of keto group to hydroxyl group (phenyllactic acid). No phenyllactic acid derivatives were observed from the supplementation of these two photophore-containing l-phenylalanine derivatives. Phenylacetaldehyde intermediates were oxidized to phenylacetic acid in the same metabolic pathway as unsubstituted l-phenylalanine. However, the bicyclic skeleton in 4-benzoyl-l-phenylalanine promoted the reduction of an aldehyde to a hydroxyl group, which was only observed in the l-tryptophan metabolic pathway. 4-Benzoyl-l-phenylalanine was metabolized to phenylethanol and phenylacetic acid. On the other hand, 4-[3-(trifluoromethyl)-3*H*-diazirin-3-yl] substitution represents an electron-withdrawing group [13]. This property promoted the reduction of the α-keto group, which was preferred over decarboxylation, and afforded phenyllactic acid derivatives as the sole product. 4-Nitro-l-phenylalanine, which has an electron-withdrawing group, was also metabolized to a phenyllactic acid skeleton.

## 3. Experimental

### 3.1. General Procedures

NMR spectra were measured on JEOL EX-280 or Bruker AMX500 spectrometers. All solvents were of reagent grade and purified using the appropriate methods. ESI-TOF-MS and FD-MS data were obtained with a Waters UPLC ESI-TOF and JEOL JMS-T100GCV mass spectrometer, respectively. Chiral HPLC for synthetic photoreactive l-phenylalanine derivatives was achieved using a Chirobiotic T column (Astec, Bellefonte, PA, USA) in 10% CH_3_OH-H_2_O.

### 3.2. Synthesis of 4-azido-l-phenylalanine(**1a**, Scheme 3)

A mixed solution of concentrated HNO_3_ (60%) and concentrated H_2_SO_4_ (1.4:1.1 v/v) was prepared and chilled to 10 °C. Then, a portion of this solution (3.5 mL) was added dropwise under stirring to a solution of l-phenylalanine (4.139 g, 25.1 mmol) in H_2_SO_4_ (98%, 12.5 mL). The reaction mixture was stirred at 10 °C for 2.5 h. The reaction solution was then adjusted to pH 5 with NH_4_OH. The pale yellow precipitate formed was collected by filtration, washed with a small volume of water and CH_3_CN, and then dried to yield 4-nitro-l-phenylalanine as a yellow powder (4.748 g, 90%). 4-Nitro-l-phenylalanine (2.006 g, 9.54 mmol) was suspended in water (35 mL) and subjected to hydrogenation at room temperature for 3 h in the presence of 0.2 g of 5% palladium-barium sulfate. The catalyst was filtered through a Celite pad, and the pale brown filtrate was concentrated. The residue was washed with CH_3_CN to afford a pale brown mass (1.460 g, 85%). No further purification was performed for the following step. The 4-amino-l-phenylalanine derivative (0.060 g, 0.33 mmol) was dissolved in 6N HCl (4 mL). Sodium nitrate in water (0.030 g/0.5 mL) was added at 0 °C. The reaction mixture was stirred at the same temperature for 40 min and diluted with 6N HCl (0.25 mL). Subsequently, sodium azide in water (0.033 g/0.75 mL) was added at 0 °C. The reaction mixture was stirred at same temperature for 5 min, and then warmed to room temperature for 1 h, then concentrated. The residue was reprecipitated from CH_3_CN to afford a pure colorless amorphous mass (0.0435 g, 64%). Analytical and spectroscopic data were identical to those reported in the literature [10].

### 3.3. Synthesis of 4-benzoyl-l-phenylalanine (**2a**, Scheme 3)

Trifluoromethanesulfonic acid (0.5 mL) was added to l-phenylalanine (16.5 mg, 0.1 mmol) in a tube fitted with a screw cap and PTFE-faced rubber liner. After the solution became homogeneous, benzoyl chloride (8 equivalents) was added at 0 °C. The reaction mixture was stirred at room temperature for 12 h, then poured into cold water and ethyl acetate. The organic layer was washed with saturated NaCl, and dried over MgSO_4_, then filtrated. The filtrate was concentrated and the residue was subjected to silica column chromatography (ethyl acetate-MeOH-H_2_O = 8:1:0.5) to afford colorless amorphous mass (7.5 mg, 28%, 98% ee). ^1^H-NMR (CD_3_OD): δ 7.76 (d, 2H, *J* = 8.6 Hz, Ar), 7.75 (d, 2H, *J* = 8.6 Hz, Ar), 7.64 (t, 1H, *J* = 7.4 Hz, Ar), 7.52 (t, 2H, *J* = 7.4 Hz, Ar), 7.47 (d, 2H, *J* = 8.0 Hz, Ar), 3.83 (m, 1H, H_α_), 3.35 (m, 1H, H_β_), 3.09 (m, 1H, H_β_).

**Scheme 3:**
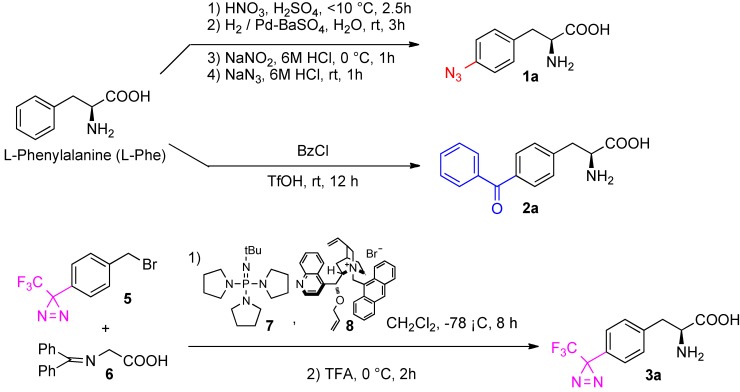
Preparations of *p*-photophore substituted l-phenylalanine derivatives.

### 3.4. Synthesis of 4-[3-(trifluoromethyl)-3H-diazirin-3-yl]-l-phenylalanine (**3a**, Scheme 3)

The synthesis of title compound was performed according to our previous asymmetric synthesis protocols with slightly modifications [11,12]. Briefly, the diazirinyl benzyl bromide (**5**, 30.0 mg, 0.10 mmol) was asymmetrically condensed with diphenyliminoglycine *t*-butyl ester (**6**, 40.8 mg, 0,14 mmol) in the presence of catalytic cinchonidium salt (**8**) and phosphazene base BTPP (**7**) at −78 °C. The reaction mixture was directly deprotected with TFA to minimize losses due to purification. The reaction mixture was subjected to silica column chromatography (ethyl acetate: MeOH: H_2_O = 8:1:0.5) to afford colorless solid (7.7 mg, 28%, 98% ee). ^1^H-NMR (CD_3_OD): δ 7.47 (d, 2H, *J* = 7.6 Hz, Ar), 7.26 (d, 2H, *J* = 7.6 Hz, Ar), 3.84 (m, 1H, H_α_), 3.40 (m, 1H, H_β_), 3.10 (m, 1H, H_β_).

### 3.5. Culture Medium and Growth Conditions

Culture medium and growth conditions were described previously [8]. Phenylalanine derivatives in DMSO: H_2_O (1:1; 75 mg/L) were filtered using a filter unit and then supplemented. The inoculated mixture formed the standing culture under conditions of 28 °C and darkness for 7 days.

### 3.6. Extraction of Metabolites

After incubation, cultured media were centrifuged at 3,500 × *g* for 10 min. The supernatant was acidified with aqueous HCl to pH 2.5 and it was then extracted with equal volumes of ethyl acetate three times. The organic layer was dried over MgSO_4_, filtered, and concentrated to afford crude extracts. The crude extracts were subjected to silica column chromatography (pre-washed with methanol and ethyl acetate-methanol-water in a ratio of 4:1:1) to afford pure metabolites.

### 3.7. 2-(4-Azidophenyl)acetic Acid (**1b**)

Inoculation of 4-azido-l-phenylalanine (**1a**, 5.0 mg) afforded a phenylacetic acid type metabolite (2.3 mg). The analytical data was identical with previous reports [14]. ^1^H-NMR (CDCl_3_): δ 7.28 (d, 2H, *J* = 7.8 Hz, Ar), 7.01 (d, 2H, *J* = 7.8 Hz, Ar), 3.65 (m, 2H, 2-H), IR (film): 2150 cm^−1^, ESI-MS (negative): *m/z* 176 [M−H]^−^. 

### 3.8. 2-(4-Benzoylphenyl)acetic acid (**2b**) and (4-(2-hydroxyethyl)phenyl)(phenyl)methanone (**2c**)

Inoculation of 4-benzoyl-l-phenylalanine (**2a**, 10.0 mg) afforded phenylacetic acid (2.3 mg) and phenylethanol type metabolites (2.2 mg). Analytical and spectroscopic data were identical to those reported in the literature [15].

*2-(4-Benzoylphenyl)acetic acid* (**2b**) ^1^H-NMR (CDCl_3_): δ 7.80 (m, 4H, Ar), 7.58 (d, 1H, *J* = 7.8 Hz, Ar), 7.49 (d, 2H, *J* = 7.8 Hz, Ar), 7.42 (d, 2H, *J* = 7.8 Hz, Ar), 3.68 (m, 2H, 2-H), FD-MS: *m/z* 240 [M]^−^. 

*(4-(2-Hydroxyethyl)phenyl)(phenyl)methanone* (**2c**) ^1^H-NMR (CDCl_3_): δ 7.77 (m, 4H, Ar), 7.60 (d, 1H, *J* = 7.8 Hz, Ar), 7.48 (d, 2H, *J* = 7.8 Hz, Ar), 7.36 (d, 2H, *J* = 7.8 Hz, Ar), 3.93 (t, 2H, *J* = 7.8 Hz, CH_2_), 2.97 (m, 2H, *J* = 7.8 Hz, CH_2_), FD-MS: *m/z* 226 [M]^−^.

### 3.9. 2-Hydroxy-3-[4-[3-(trifluoromethyl)-3H-diazirin-3-yl]phenyl]propanoic Acid (**3b**)

Inoculation of 4-[3-(trifluoromethyl)-3*H*-diazirin-3-yl]-l-phenylalanine (**3a**, 10.0 mg) afforded a phenyllactic acid type metabolite (2.3 mg). ^1^H-NMR (CD_3_OD): δ 7.38 (d, 2H, *J* = 7.8 Hz, Ar), 7.12 (d, 2H, *J* = 7.8 Hz, Ar), 4.20 (m, 1H, 2-H), 3.46 (m, 1H, 3-Ha), 3.15 (m, 1H, 3-Hb), ESI-MS (negative): *m/z* 273 [M−H]^−^, 246 [M−N_2_−H]^−^. λ_max_ (ε) (CH_3_OH) 355 (380).

### 3.10. 2-Hydroxy-3-(4-nitrophenyl)propanoic Acid (**4d**)

Inoculation of 4-nitro-l-phenylalanine (**4a**, 15.0 mg) afforded a phenyllactic acid type metabolite (2.3 mg). ^1^H-NMR (CD_3_OD): δ 8.15 (d, 2H, *J* = 7.8 Hz, Ar), 7.51 (d, 2H, *J* = 7.8 Hz, Ar), 4.37 (m, 1H, 2-H), 3.56 (m, 1H, 3-Ha), 3.03 (m, 1H, 3-Hb), ESI-MS (negative): *m/z* 210 [M−H]^−^. 

## 4. Conclusions

Inoculation of l-phenylalanine afforded different metabolites in *Klebsiella* sp CK6 compared with l-tryptophan. Both lactic acid (ILA and PLA) and acetic acid (IAA and PAA) skeletons are common structural units between l-phenylalanine and l-tryptophan. However, no ethanol skeleton was detected after the inoculation of l-phenylalanine. These results indicated that a bicyclic (indole) moiety was important for the enzymatic reduction of acetaldehyde moiety under TOL-type metabolism conditions. The stereochemistry of the precursor phenylalanine preferred the L-configuration rather than the d-configuration. Based on the analysis of phenylalanine metabolites, the photophore-substituted l-phenylalanines participated in the metabolic pathway. The photochemical properties of the photophores—phenyl azide, benzophenone, and trifluoromethylphenyldiazirine—have been compared to assess the success of photoaffinity labeling to target biomolecules. There is no doubt that photophore-introduced phenylalanines afforded the same metabolites, and, in addition, most of the photoreactive derivatives acted in an identical manner as phenylalanine. This study presents the first observations of a comprehensive metabolite analysis of different photophores containing l-phenylalanine derivatives and the different metabolites afforded by each photophore. These results indicated that there is possibility for different photoaffinity labeling of metabolic enzymes, which can be achieved using different photophore-containing phenylalanine derivatives.
